# Exploring serum and immunoglobulin G *N*-glycome as diagnostic biomarkers for early detection of breast cancer in Ethiopian women

**DOI:** 10.1186/s12885-019-5817-8

**Published:** 2019-06-17

**Authors:** Abrha G. Gebrehiwot, Daniel Seifu Melka, Yimenashu Mamo Kassaye, Tufa Gemechu, Wajana Lako, Hiroshi Hinou, Shin-Ichiro Nishimura

**Affiliations:** 1 Faculty of Advanced Life Science and Graduate School of Life Science, Hokkaido University, N21, W11, Kita-ku, Sapporo, 001-0021 Japan; 2 Department of Biochemistry, School of Medicine, Addis Ababa University, Addis Ababa, Ethiopia; 3 Department of Pathology, School of Medicine, Addis Ababa University, Addis Ababa, Ethiopia

**Keywords:** Breast cancer, Early stage biomarker, N-glycan, Glycoblotting, Serum, IgG

## Abstract

**Background:**

Alterations in protein glycosylation patterns have potentially been targeted for biomarker discovery in a wide range of diseases including cancer. Although there have been improvements in patient diagnosis and survival for breast cancer (BC), there is no clinically validated serum biomarker for its early diagnosis. Here, we profiled whole serum and purified Immunoglobulin G (IgG) fraction *N*-glycome towards identification of non-invasive glycan markers of BC.

**Methods:**

We employed a comprehensive glycomics approach by integrating glycoblotting-based glycan purification with MALDI-TOF/MS based quantitative analysis. Sera of BC patients belonging to stages I-IV and normal controls (NC) were collected from Ethiopian women during 2015–2016. IgG was purified by affinity chromatography using protein G spin plate and further subjected to glycoblotting for glycan release. Mass spectral data were further processed and evaluated rigorously, using various bioinformatics and statistical tools.

**Results:**

Out of 35 *N*-glycans that were significantly up-regulated in the sera of all BC patients compared to the NC, 17 complex type *N*-glycans showed profound expression abundance and diagnostic potential (AUC = 0.8–1) for the early stage (I and II) BC patients. Most of these glycans were core-fucosylated, multiply branched and sialylated structures, whose abundance has been strongly associated with greater invasive and metastatic potential of cancer. *N*-glycans quantified form IgG confirmed their abundance in BC patients, of which two core-fucosylated and agalactosylated glycans (*m/z* 1591, 1794) could specifically distinguish (AUC = 0.944 and 0.921, *p* ≤ 0.001) stage II patients from NC. Abundance of such structural features in IgG is associated with a decrease in its immunosuppressive potential towards tumor cells, which in part may correlate with the aggressive nature of BC commonly noticed in black population.

**Conclusions:**

Our comprehensive study has addressed for the first time both whole serum and IgG *N*-glycosylation signatures of native black women suffering from BC and revealed novel glyco-biomarkers with marked overexpression and distinguishing ability at early stage patients. Further studies on direct identification of the intact glycoproteins using a glycoprteomics approach will provide a deeper understanding of specific biomarkers towards their clinical utility.

**Graphical abstract:**

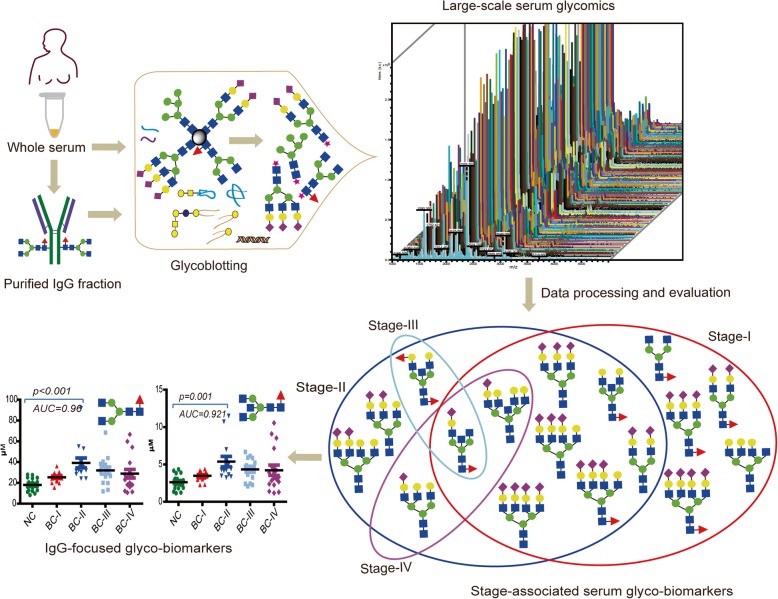

**Electronic supplementary material:**

The online version of this article (10.1186/s12885-019-5817-8) contains supplementary material, which is available to authorized users.

## Background

Based on a recent report, breast cancer (BC) has become the most common malignancy and the leading cause of cancer death among women globally [1]. In clinical settings, the disease progression is classified into four major stages (stages I-IV) based on pathological assessments named as TNM system (‘T’ for the size of the tumor; ‘N’ for the involvement of lymph nodes; ‘M’ for metastasis to other parts of the body)**.** Distant metastases account for over 90% of breast cancer deaths with bone, lung, liver, and brain ranked as the vital target organs for breast cancer metastasis [2, 3]. If diagnosed at an early stage, BC has a high rate of survival (5-year survival rate after diagnosis is 99% when the tumor is still localized, but 27% for distant-stage disease) [2]. The current method for early-stage diagnosis of BC is mammography. Nevertheless, in addition to its inaccurate diagnosis, many patients do not receive mammograms regularly due to lack of access to the facility, high cost, procedural discomfort, and perceived risks of radiation exposure [4]. Consequently, there has been an increasing interest in the development and validation of serum-based non-invasive biomarkers whose alteration in the serum precedes the appearance of a malignancy, of which there is currently none [5].

Glycosylation, a complex post-translational modification involving covalent attachment of sugars to proteins or lipids, has recently received attention as a key component of cancer progression. Hence, unique expression or quantitative alterations in tumor-associated glycans can provide novel diagnostic and therapeutic targets [6, 7]. The fact that most human serum proteins are heavily glycosylated makes them a potential reservoir of glycans released from tissues and cells, reflecting their physiological and pathological states [8, 9]. In cancer progression, serum *N*-glycan alterations have been associated with proliferation, invasion, metastasis, aggressiveness, angiogenesis, and immune regulation of tumor cells [10].

With recent remarkable improvements in analytical tools, mass spectrometry is a widely used method to analyze glycans, proteins, or glycoproteins for clinical biomarker discovery [11]. In this regard, previous studies have suggested *N*-glycan biomarkers for a wide range of cancer types [12–19]. However, the glycomic studies performed to date on breast cancer were focusing either on animal models, cultured cell lines, a small number of human patients, or didn’t employ the current state of methods for glycan purification and quantitative analysis from multiple types of biological samples [19–24]. Apart from total serum *N*-glycome, recent studies have also illustrated the association of altered glycosylation patterns of immunoglobulin G (IgG) with the progression of several tumors [25–27]. Using a recently developed novel glycoblotting method [28], we could screen serum *N*-glycan biomarkers for different pathological states including cancer, neurodegenerative diseases, and other eye, kidney, and arthritis related disorders [16–18, 29–34].

Driven by our advanced glycoblotting technology and the unmet needs for better molecular biomarkers and drug targets, this study was aimed to explore the potentials of *N*-glycans released from patient serum glycoproteins and from purified IgG fraction towards discovery of clinical biomarker for breast cancer. For this purpose, our study population were from Ethiopia where breast cancer incidence has been increasing drastically in younger women, while inadequate and ineffective control measures are increasing the mortality rate.

## Methods

### Study population and sample collection

Serum samples were collected from 115 BC female patients clinically belonged to stages I-IV and as normal controls (NC), serum samples from 33 gender, ethnic, and age matched healthy volunteers were obtained. All study subjects were Ethiopians and serum collection was performed during 2015–2016 in the Oncology Center of Black Lion Specialized Teaching Hospital (the largest and the only hospital with cancer treatment center in the country) of Addis Ababa University, Ethiopia. Informed consent had been signed and obtained from all study participants. The study was performed in accordance with the ethical standards of the Declaration of Helsinki on approval by the ethical review boards of Addis Ababa University, School of Medicine, Ethiopia and Hokkaido University, Faculty of Advanced Life Sciences, Japan. After freezed in plastic vials at − 80 °C and carefully packed with dried CO_2_ in foam box, serum samples were immediately transported by airplane and reached Japan within 24 h.

### Reagents and materials

Ammonium bicarbonate 99% (ABC), 1-propanesulfonic acid, 2-hydroxyl-3-myristamido (PHM), 3-Methyl-1-*p*-tolyltriazene (MTT), and disialyloctasaccharide were purchased from Tokyo Chemical Industry Co., Ltd. (Tokyo, Japan). Peptide *N*-glycosidase F (PNGase F) was purchased from New England Biolabs^R^ Inc. (Ipswich, Massachusetts, USA), whereas BlotGlyco H beads were purchased from Sumitomo Bakelite, Co. Ltd. (Tokyo, Japan). Dithiothreitol (DTT), iodoacetamide (IAA), *O*-benzylhydroxylamine hydrochloride (BOA), α-cyano-4-hydroxycin-namic acid (CHCA) and trypsin were from Sigma-Aldrich, Inc. (St. Louis, MO, USA). Other reagents and solvents were obtained from Wako Pure Chemical Industries, Ltd. (Tokyo, Japan), unless otherwise stated. Protein G spin plate was purchased from Thermo Scientific. SweetBlot™ (automated glycan processing and incubating machine) was from System Instruments Co., Ltd. (Hachioji, Japan). Multi Screen Slvinert^R^ filter plates were purchased from Millipore Co., Inc. (Tokyo, Japan). All mass quantifications were done using MALDI-TOF/MS (Ultraflex III, Brukers Daltonics, Germany).

### Immunoglobulin G purification from whole serum

IgG was purified from serum by affinity chromatography using 96-well protein G spin plate (Thermo Scientific) that is applied by fixing on another wash/collection plate as per the manufacturer’s protocol. First, 50 μL of serum was diluted 4 times with binding buffer (PBS, pH 7.2) and applied to the wells. The plate assembly was placed on a plate shaker and incubated for 30 min with moderate agitation, followed by centrifugation at 1000×g for 1 min with discarding the flow-through. The resin on the purification plate was washed 4 times by adding 300 μL of PBS to each well and then centrifuging at 1000×g for 1 min, discarding the flow-through each time. Before elution, 20 μL of neutralization buffer (1 M Tris-HCl, pH 9) was added to each well of new collection plate to maintain IgG stability. Subsequently, IgG was eluted by adding 200 μL of 0.1 M formic acid, pH 2.7 and centrifuging at 1000×g for 1 min. This step was repeated twice for complete elution. After confirming the purity of the IgG fraction by SDS-PAGE, it was dried using speed vac and then reconstituted in 50 μL of pure water to be applied for glycan release and purification by glycoblotting.

### Enzymatic release of *N*-glycans and glycoblotting

**Fig. 1:**
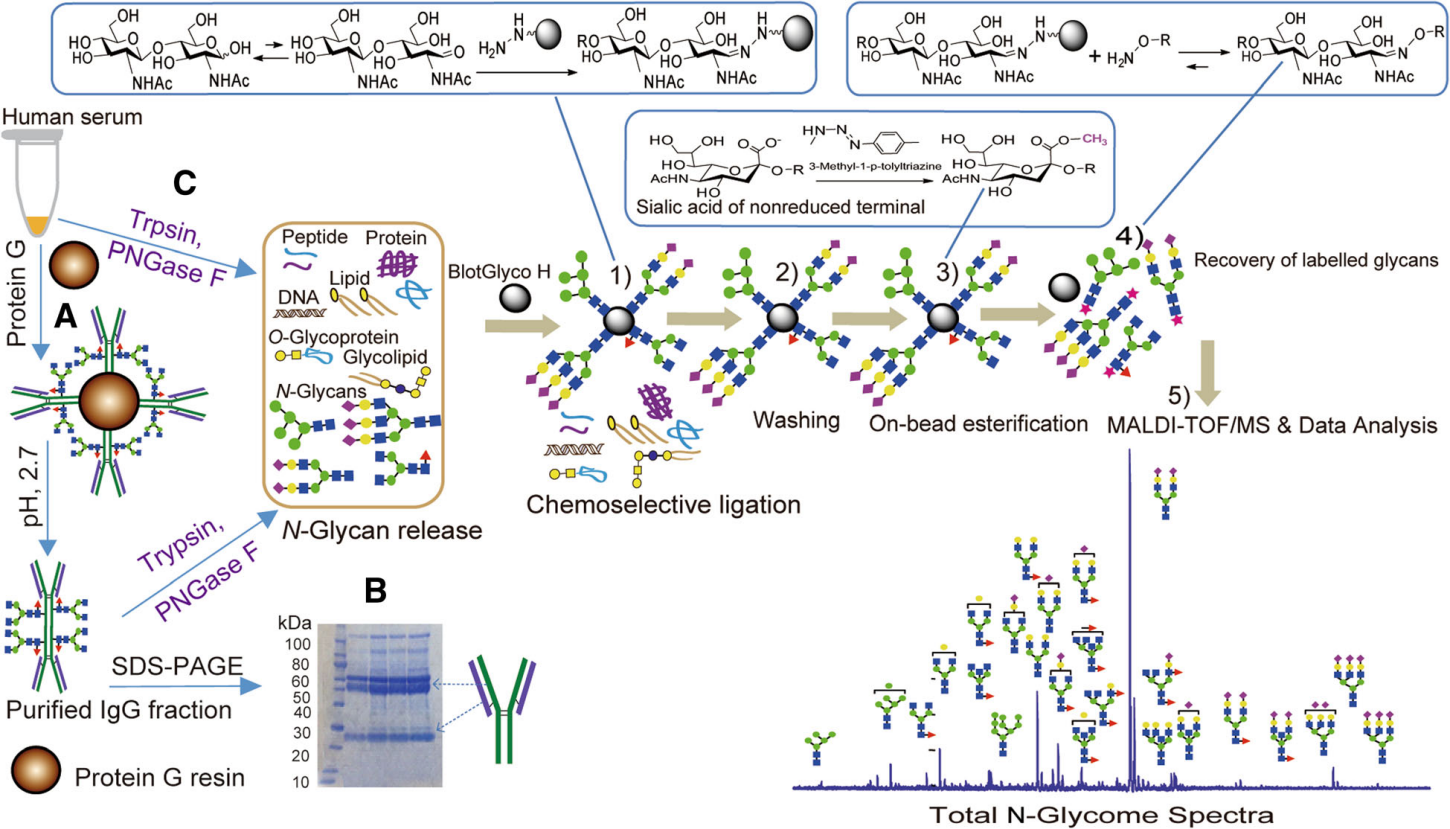
Schematic workflow of glycoblotting-based MALDI-TOF/MS analysis of *N*-glycomes derived from whole serum and purified IgG fraction. The major steps include: **a** Purification of IgG fraction from whole serum using protein G resin, **b** confirmation of IgG by SDS-PAGE, **C**) Enzymatic release of glycans directly from serum or from IgG fraction, 1) Chemoselective capturing of reducing sugars onto a hydrazide-functionalized BlotGlycoH beads, 2) washing to remove all impurities, 3) On-bead methyl esterification of sialic acid residues, 4) Recovery of BOA-labeled glycans, 5) MALD-TOF/MS and data analysis

All the procedures for *N*-glycan release, purification, labeling, and spotting were performed in the SweetBlot 7 automated system (all-in-one approach) as previously described [28, 35–37]. Accordingly, whole serum or IgG fraction purified from each sample was pretreated enzymatically for producing oligosaccharides carrying reducing terminal that will enable them to chemically ligate with hydrazide-functionalized BlotGlyco^H^ beads during the subsequent glycoblotting process. Hence, 10 μL of whole serum or IgG fraction was auto-transferred into a 96 well polymerase chain reaction (PCR) plate and was dissolved with freshly prepared 0.33 M Ammonium bicarbonate (ABC) containing 120 mM 1, 4-dithiothreitol (DTT) and 0.4% of 1-propanesulfonic acid, 2-hydroxyl-3-myristamido (PHM) in 10 mM ABC. As an internal standard, 12 μL of 60 μM disialyloctasaccharide was also added and mixed in each well, enabling us to perform eventual absolute quantification of individual glycan level. Solubilized proteins were reduced by incubation at 60 °C for 30 min, followed by alkylation with 10 μL of 123 mM iodoacetamide in the dark at room temperature for 1 h. The mixture was then digested with 400 U of trypsin in 1 mM HCl at 37 °C for 2 h, and subsequently heated at 90 °C for 10 min to inactivate the enzyme. Soon after being cooled to room temperature, free *N*-glycans were released using 2 U of Peptide *N*-Glycosidase F (PNGase F) and incubating at 37 °C for 6 h. In the glycoblotting-based strategy (diagrammed in Fig. 1), 250 μL of BlotGlyco H bead (Sumitomo Bakelite Co., Ltd., 10 mg/mL suspension with water) was placed into a well of a Multi Screen Solvinert filter plate (Millipore), by vacuuming to remove the water. 20 μL of PNGase F digested mixture containing released *N*-glycans was mixed with the bead in each well, followed by the addition of 180 μL of 2% acetic acid (AcOH) in acetonitrile (ACN). To capture the *N*-glycans in sample mixtures specifically onto beads via reversible hydrazone bonds, the plate was incubated at 80 °C for 45 min. Then, the beads were washed twice with each 200 μL of 2 M guanidine-HCl in ABC, water, and 1% triethyl amine in methanol. Unreacted hydrazide functional groups on beads were capped by incubation with 10% acetic anhydride in MeOH for 30 min at room temperature. After removing the solution by vacuum, the beads were successively washed twice with each 200 μL of 10 mM HCl, MeOH, and dioxane. To stabilize sialic acids, on-bead methyl esterification of carboxyl groups in sialic acids was carried out by incubation with fresh 100 mM 3-methyl-1-*p*-tolyltriazene (MTT) in dioxane at 60 °C for 90 min. Each well was then washed twice using 200 μL of dioxane, water, methanol, and water again. The glycans captured on beads were subjected to the *trans*-iminization reaction with 20 μL of 50 mM *O*-benzyloxyamine hydrochloride (BOA) and with mild acid hydrolysis of hydrazone bond using 180 μL of 2% AcOH in ACN at 80 °C for 45 min. Finally, the released and BOA-tagged *N*-glycans were eluted with 75 μL of water under vaccum and applied for subsequent MALDI-TOF/MS analysis.

### MALDI-TOF/MS analysis of *N*-glycans

BOA-labeled *N*-glycans were directly dissolved with an equivalent volume of self-prepared ionic liquid matrix solution (100 mM α-cyano-4-hydroxycinnamic acid diethylamine salt in MeOH: H2O: DMSO: 10 mM NaOH: = 50:39:10:1), after which 2.5 μL of the sample-matrix mixture was spotted onto MTP 384 target plate (polished steel TF, Bruker Daltonics). Each sample was spotted into 4 replicates to ensure reproducibility of the experiment. After being dried to crystallize the spots, *N*-glycans were analyzed using Ultraflex III MALDI-TOF/MS (Bruker Daltonics, Germany) by operation in positive-ion reflector mode, typically summing 1, 000 laser shots for each spot.

### Data processing and evaluation

The acquired MALD-TOF/MS spectra were further processed and evaluated using FlexAnalysis v. Three Software (Bruker Daltonics, Germany) and Microsoft Excel. *N*-glycans were selected based on their quantitative reproducibility after evaluation using the calibration curve of serially diluted human serum standards. The intensity of monoisotopic peak of each *N*-glycan was normalized using 60 μM of an internal standard (Disialyloctasaccharide, produced by Tokyo Chemical Industry) and this normalized data was used for further statistical analysis and quantitative comparison. Structural compositions of *N*-glycans were speculated using GlycoMod Tool (http://br.expasy.org/tools/glycomod/).

### Statistical analysis

Data for *N*-glycan abundance were analyzed using SPSS software. Multiple comparisons among NC and clinical stage groups were performed using a bonferroni one-way analysis of variance (ANOVA) while comparison between two (NC vs whole BC) groups was performed using Independent-Samples T-Test. Mean value differences were considered to be significant at 95% confidence interval (*p* ≤ 0.05). The diagnostic potential of significantly differed individual glycans or glyco-subclasses was further analyzed by receiver operator characteristic (ROC) test. Area under the curve (AUC) value generated from ROC test was used to assess the diagnostic accuracy of potential glycan biomarkers. Accordingly, AUC values of 0.9–1, 0.8–0.9, 0.7–0.8, and < 0.7 indicate a “highly accurate”, “accurate”, “moderately accurate”, and uninformative test, respectively. We also used Graph Pad prism 5 software to show the data distribution in scatter dot plot.

## Results

### Patient characteristics

Descriptive information on the selected study participants is presented in Table 1. All the data were collected during the time of diagnosis and individuals who were pregnant, were receiving any treatment, or were with a medical history of disease which can affect the glycan profile, were excluded from the study. Age and gender were matched among the clinical groups. However, with the majority of the young patients, still the control group was found to be slightly younger (Table 1, A and B). Overall, the relatively younger age of the study population (uncommon in prior cancer studies) can be taken as an advantage in order to rule out the impact of aging on glycan profile.

**Table 1:** Demographic characteristics of normal controls (NC) and breast cancer (BC) patients of different stages

Status	Number (n)	*A. Mean ± SD*			*B. Number of subjects per age range*				
		Age at diagnosis	BMI (kg/m^2^)	Age at Menarche	20–35	36–45	46–55	56–65	> 65
NC	24	31.54 ± 7.17	22.17 ± 2.97	14.29 ± 1.46	16	7	1		
BC-I	19	42.74 ± 14.04	22.14 ± 2.52	15.32 ± 1.53	8	3	3	5	
BC-II	23	43.7 ± 12.76	22.01 ± 3.01	15 ± 1.38	6	10	3	3	1
BC-III	25	41.4 ± 12.02	21.78 ± 3.02	14.48 ± 1.69	9	6	8	1	1
BC-IV	28	43.25 ± 13.84	22.89 ± 2.92	14.39 ± 1.4	10	8	6	2	2
Total (N)	119				49	34	21	11	4

*NC* normal control, *BC* breast cancer, *SD* standard deviation, *BMI* body mass index

### Quantitative reproducibility test

**Fig. 2:**
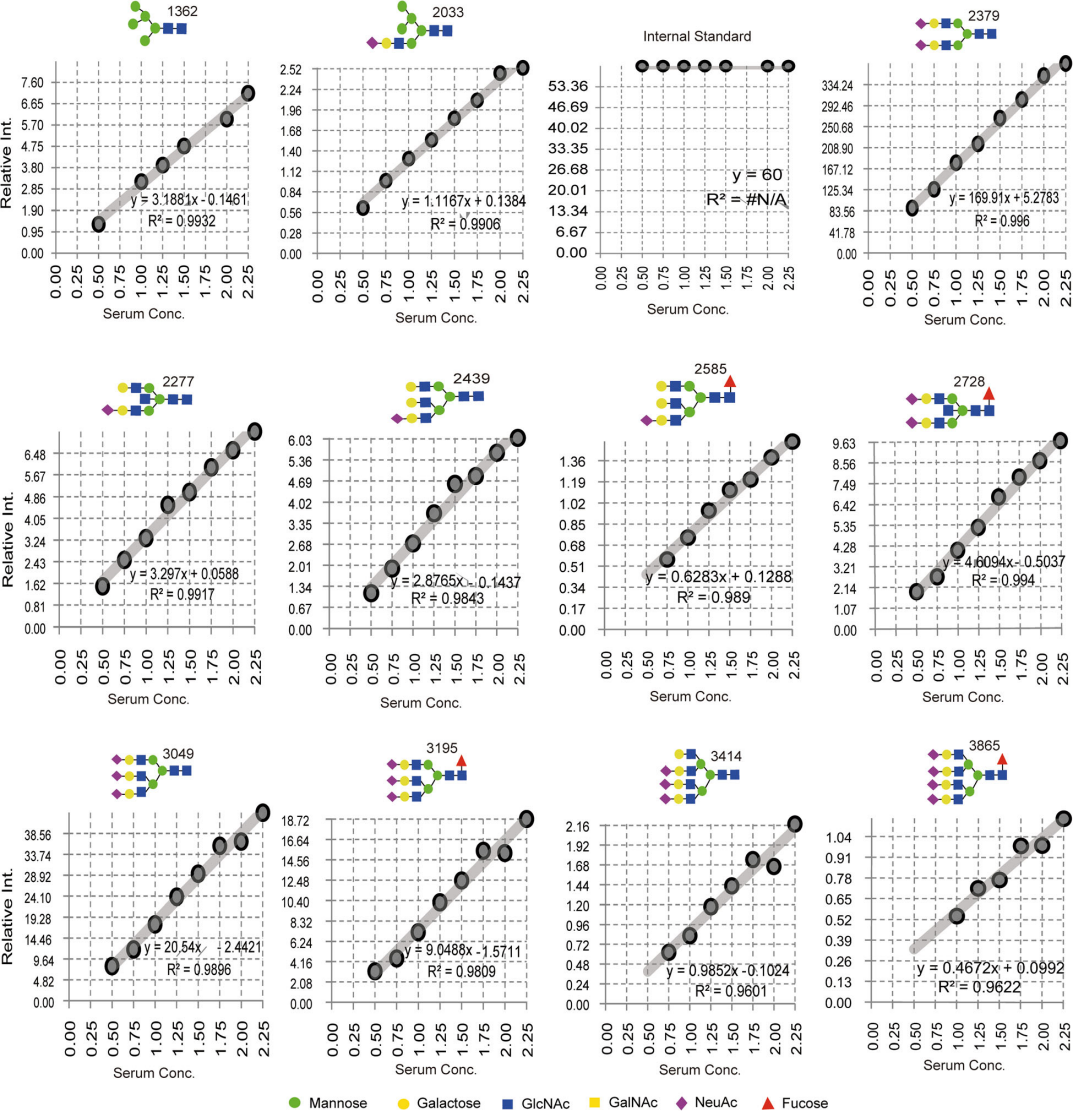
Human serum standard calibration curve showing quantitative reproducibility of some common *N*-glycans

Since each serum or IgG sample was spotted in four replicates, four normalized spectral data for each sample were averaged before statistical analysis. Before selection of the detected *N*-glycans, each peak was evaluated for its quantitative reproducibility using serially diluted standard human serum samples that had been experimented in the same plate beside the patient samples. The peak area of each glycan detected in the dilution series (0.5×, 0.75×, 1×, 1.25×, 1.5×, 1.75×, 2×, and 2.25×) was normalized using known concentration of internal standard, after which a standard calibration curve was plotted for each glycan as shown in Fig. 2. Quantitative reliability was then judged based on minimum outlier scores, good slope linearity, and significant level of the correlation coefficient across the standard curve. Accordingly, glycan peaks that meet these criteria were selected and considered for further statistical analysis in the main study samples.

### Total serum and IgG *N*-glycan profiles

**Fig. 3:**
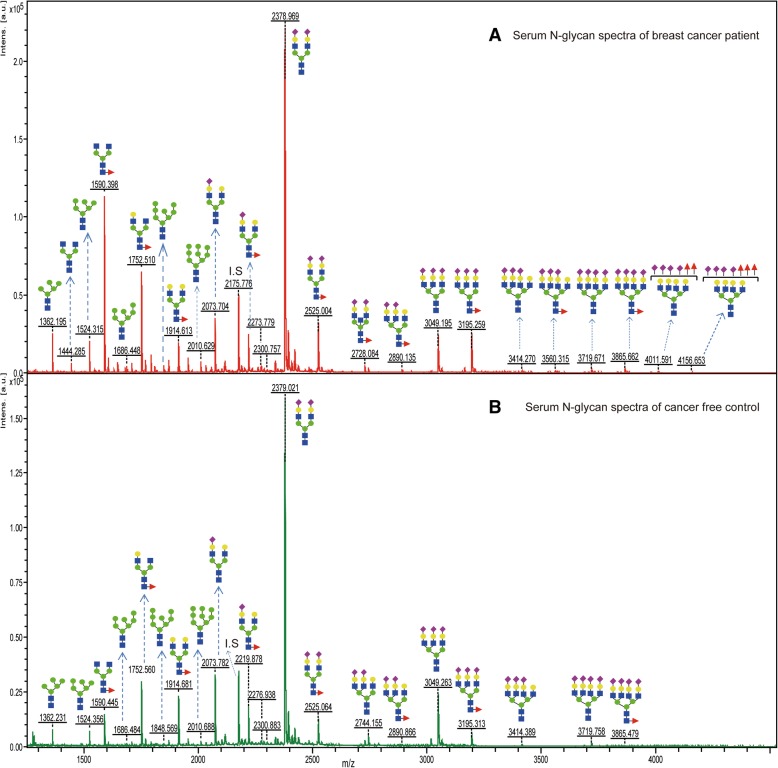
Representative MALDI-TOF/MS spectra of serum *N*-glycans derived from individuals with BC (**a**) and free of BC (**b**). The two glycans with m/z of 4011.591 and 4156.663 were not considered for quantitative comparison since their detection was not quantitatively reproducible in all patients

**Fig. 4:**
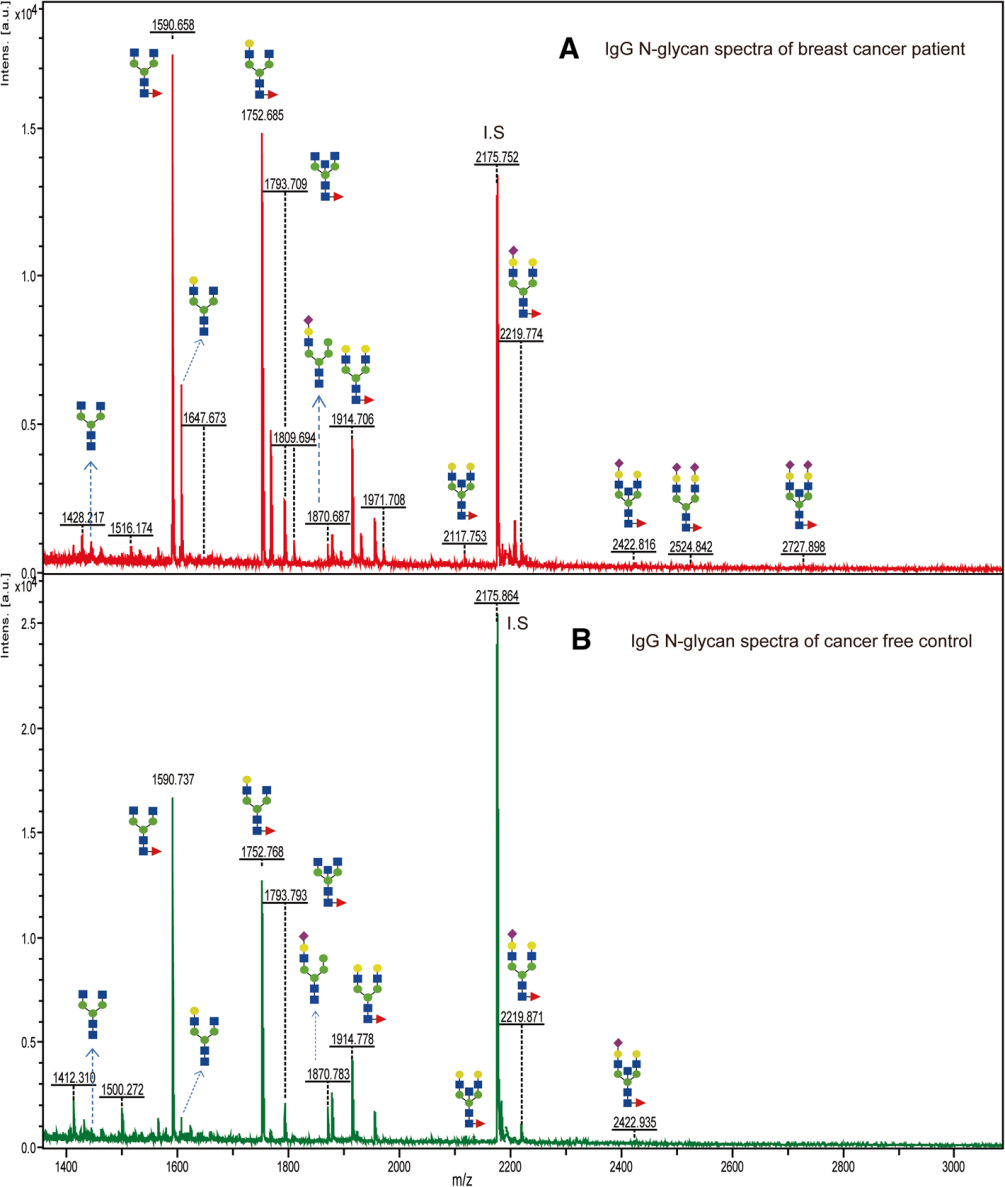
Representative MALDI-TOF/MS spectra of IgG *N*-glycans from individuals with BC (**a**) and free of BC (**b**)

Using an integrated protocol for glycan release and purification, coupled with MALDI-TOF/MS analysis, 46 *N*-glycans (Table 2) could be detected in the serum of entire study samples. 37 (80.43%) of the detected *N*-glycans belong to complex type whereas high-mannose and hybrid types comprise 5 (10.87%) and 4 (8.9%), respectively. About one-third of these serum glycans were detected in the IgG fraction as well (indicated with “+” sign in Table 2). The large-scale mass spectra of serum *N*-glycans (Additional file 1: Figure S1) have shown quite reproducible patterns with nonnegligible differences in the peak intensity of many glycans (shown in Fig. 3) between patients and controls. Similarly, the collective mass spectra for IgG *N*-glycome is shown in Additional file 2: Figure S2 from which recognizable variations in terms of peak intensity and detection patterns between BC patients and controls can be seen in the individual mass spectrum (Fig. 4).

**Table 2:** Estimated composition of 46 *N*-linked glycans identified from human serum glycoproteins

Pea#	*m/z*	Glycan composition	Type	In IgG
1	1362.48109	(Hex)2 + (Man)3 (GlcNAc)2	High-Mannose	
2	1444.53419	(HexNAc)2 + (Man)3 (GlcNAc)2	Complex	**+**
3	1524.53392	(Hex)3 + (Man)3 (GlcNAc)2	High-Mannose	**+**
4	1565.56047	(Hex)2 (HexNAc)1 + (Man)3 (GlcNAc)2	Hybrid	**+**
5	1590.59210	(HexNAc)2 (Deoxyhexose)1 + (Man)3 (GlcNAc)2	Complex	**+**
6	1606.58702	(Hex)1 (HexNAc)2 + (Man)3 (GlcNAc)2	Complex	**+**
7	1686.58675	(Hex)4 + (Man)3 (GlcNAc)2	High-Mannose	
8	1708.61871	(Hex)1 (HexNAc)1 (NeuAc)1 + (Man)3 (GlcNAc)2	Complex	
9	1752.64493	(Hex)1 (HexNAc)2 (Deoxyhexose)1 + (Man)3 (GlcNAc)2	Complex	**+**
10	1768.63985	(Hex)2 (HexNAc)2 + (Man)3 (GlcNAc)2	Complex	**+**
11	1793.67148	(HexNAc)3 (Deoxyhexose)1 + (Man)3 (GlcNAc)2	Complex	**+**
12	1848.63958	(Hex)5 + (Man)3 (GlcNAc)2	High-Mannose	
13	1854.67662	(Hex)1 (HexNAc)1 (Deoxyhexose)1 (NeuAc)1 + (Man)3 (GlcNAc)2	Complex	
14	1870.67154	(Hex)2 (HexNAc)1 (NeuAc)1 + (Man)3 (GlcNAc)2	Hybrid	**+**
15	1911.69809	(Hex)1 (HexNAc)2 (NeuAc)1 + (Man)3 (GlcNAc)2	Complex	
16	1914.69776	(Hex)2 (HexNAc)2 (Deoxyhexose)1 + (Man)3 (GlcNAc)2	Complex	**+**
17	1955.72431	(Hex)1 (HexNAc)3 (Deoxyhexose)1 + (Man)3 (GlcNAc)2	Complex	**+**
18	2010.69241	(Hex)6 + (Man)3 (GlcNAc)2	High-Mannose	
19	2032.72437	(Hex)3 (HexNAc)1 (NeuAc)1 + (Man)3 (GlcNAc)2	Hybrid	
20	2057.75600	(Hex)1 (HexNAc)2 (Deoxyhexose)1 (NeuAc)1 + (Man)3 (GlcNAc)2	Complex	**+**
21	2073.75092	(Hex)2 (HexNAc)2 (NeuAc)1 + (Man)3 (GlcNAc)2	Complex	
22	2117.77714	(Hex)2 (HexNAc)3 (Deoxyhexose)1 + (Man)3 (GlcNAc)2	Complex	**+**
23	2175.78261	(Hex)2 (HexNAc)2 (NeuAc)2 + (Man)3 (GlcNAc)1	I.S	
24	2219.80883	(Hex)2 (HexNAc)2 (Deoxyhexose)1 (NeuAc)1 + (Man)3 (GlcNAc)2	Complex	**+**
25	2260.83538	(Hex)1 (HexNAc)3 (Deoxyhexose)1 (NeuAc)1 + (Man)3 (GlcNAc)2	Complex	
26	2263.83505	(Hex)2 (HexNAc)3 (Deoxyhexose)2 + (Man)3 (GlcNAc)2	Complex	
27	2276.83030	(Hex)2 (HexNAc)3 (NeuAc)1 + (Man)3 (GlcNAc)2	Complex	
28	2336.85144	(Hex)3 (HexNAc)4 + (Man)3 (GlcNAc)2	Complex	
29	2378.86199	(Hex)2 (HexNAc)2 (NeuAc)2 + (Man)3 (GlcNAc)2	Complex	
30	2422.88821	(Hex)2 (HexNAc)3 (Deoxyhexose)1 (NeuAc)1 + (Man)3 (GlcNAc)2	Complex	**+**
31	2438.88313	(Hex)3 (HexNAc)3 (NeuAc)1 + (Man)3 (GlcNAc)2	Complex	
32	2483.89335	(Hex)3 (HexNAc)1 (Deoxyhexose)1 (NeuAc)2 + (Man)3 (GlcNAc)2	Hybrid	
33	2520.93623	(Hex)1 (HexNAc)5 (NeuAc)1 + (Man)3 (GlcNAc)2	Complex	
34	2524.91990	(Hex)2 (HexNAc)2 (Deoxyhexose)1 (NeuAc)2 + (Man)3 (GlcNAc)2	Complex	
35	2581.94137	(Hex)2 (HexNAc)3 (NeuAc)2 + (Man)3 (GlcNAc)2	Complex	
36	2584.94104	(Hex)3 (HexNAc)3 (Deoxyhexose)1 (NeuAc)1 + (Man)3 (GlcNAc)2	Complex	
37	2727.99928	(Hex)2 (HexNAc)3 (Deoxyhexose)1 (NeuAc)2 + (Man)3 (GlcNAc)2	Complex	
38	2743.99420	(Hex)3 (HexNAc)3 (NeuAc)2 + (Man)3 (GlcNAc)2	Complex	
39	2890.05211	(Hex)3 (HexNAc)3 (Deoxyhexose)1 (NeuAc)2 + (Man)3 (GlcNAc)2	Complex	
40	3007.09472	(Hex)4 (HexNAc)5 (NeuAc)1 + (Man)3 (GlcNAc)2	Complex	
41	3049.10527	(Hex)3 (HexNAc)3 (NeuAc)3 + (Man)3 (GlcNAc)2	Complex	
42	3109.12641	(Hex)4 (HexNAc)4 (NeuAc)2 + (Man)3 (GlcNAc)2	Complex	
43	3195.16318	(Hex)3 (HexNAc)3 (Deoxyhexose)1 (NeuAc)3 + (Man)3 (GlcNAc)2	Complex	
44	3414.23748	(Hex)4 (HexNAc)4 (NeuAc)3 + (Man)3 (GlcNAc)2	Complex	
45	3560.29539	(Hex)4 (HexNAc)4 (Deoxyhexose)1 (NeuAc)3 + (Man)3 (GlcNAc)2	Complex	
46	3719.34855	(Hex)4 (HexNAc)4 (NeuAc)4 + (Man)3 (GlcNAc)2	Complex	
47	3865.40646	(Hex)4 (HexNAc)4 (Deoxyhexose)1 (NeuAc)4 + (Man)3 (GlcNAc)2	Complex	

Peak number 23 is an internal standard (I.S: disialyloctasaccharide). The “+” sign in the last column indicates those glycans which were also detected in IgG fraction of the samples

### Evaluation for potential serum glycan biomarker identification

**Fig. 5:**
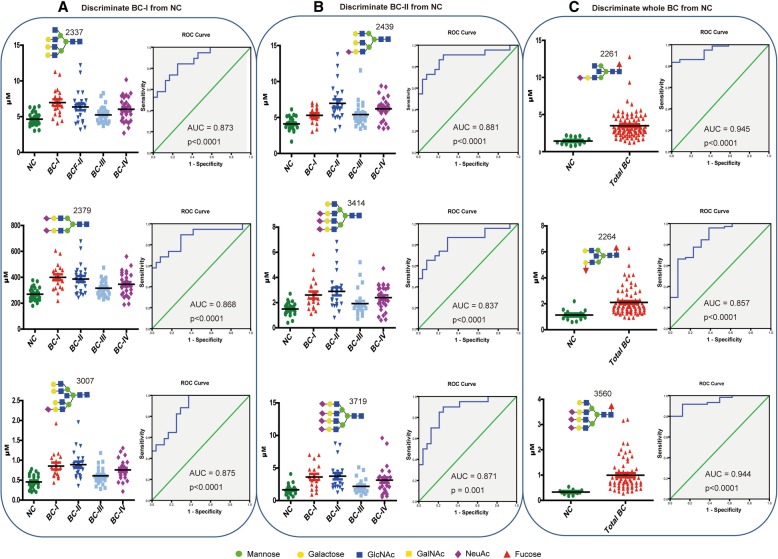
Dot plot expression and ROC curve of selected serum *N*-glycans up-regulated in BC patients comparing to NC. Area under the curve (AUC) value is displayed to indicate the discrimination power of the glycan between patients and controls **a**) Discriminate BC-I from NC; **b**) Discriminate BC-II from NC; and **c**) Discriminate whole BC from NC

**Fig. 6:**
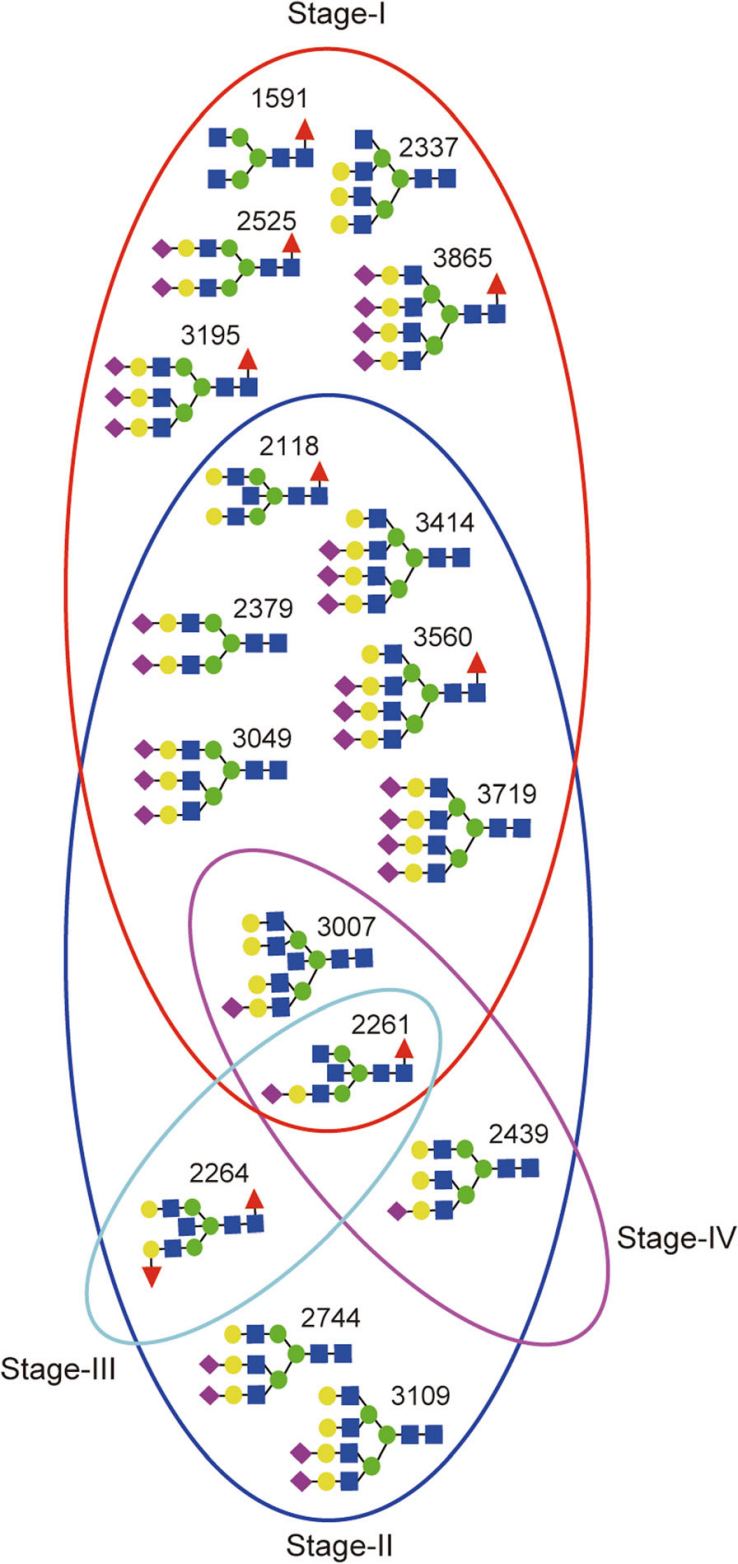
Venn diagram showing BC stage specific and overlapping *N*-glycans identified as candidate biomarkers

To identify individual glycans that showed differential expression patterns between patient and control groups, we performed Independent-Samples T-Test using the quantified data of each *N*-glycan peak. The majority of *N*-glycans exhibited up-regulated expression in which the levels of 35 *N*-glycans were significantly higher in the sera of whole BC patients compared to the controls (Additional file 3: Table S1). On the contrary, only two glycans showed reduction in the patient group in a slightly significant (m/z 1915, *p* = 0.048) and a non-significant (m/z = 2220) manner. Further multiple comparisons among the BC stages and NC group demonstrated that a substantial increase in glycan abundance was predominant at an early stage of the disease (Fig. 5). The serum glycans that had shown significant (*p* ≤ 0.05) expression alteration during ANOVA or T-test analysis were further considered for receiver operating characteristic (ROC) test. Their ability to distinguish cancer patients from controls was then evaluated based on their area under the curve (AUC) value generated from ROC analysis. As a result, 17 serum *N*-glycans with the highest diagnostic performance (detailed in Table 3) were selected as biomarkers for different BC stages and/or entire cancer patients. All these glycan structures belong to complex type *N*-glycans that include 4 bisecting, 8 core fucosylated, and 12 sialylated to a different degree with multiply branched antennas. Each of these identified glyco-biomarkers could demonstrate significant abundance with “highly accurate” (0.9–1) or “accurate” (0.8–0.9) diagnostic performance at an early stage (stage I, II, or both) or all patients, whereas no powerful biomarker was obtained exclusively at the late stages (BC-III or BC-IV) of the disease. As shown in Table 3 and Fig. 6, only 2 glycans that have bisected and fucosylated structures (m/w 2261 and 2264) for BC-III, and 3 monosialylated glycans (m/w 2261, 2439, 3007) for BC-IV met the biomarker criteria and adequately distinguished the patients from the controls.

**Table 3:** Details of serum glycan biomarkers for breast cancer stages based on ROC analysis.

*m/z*	Glycan structure	Breast cancer (BC) patients vs healthy controls										Can be Biomarker for
		BC-I (*n* = 19)		BC-II (*n* = 23)		BC-III (*n* = 25)		BC-IV (*n* = 24)		Whole BC (*N* = 95)		
		AUC	*p*	AUC	*p*	AUC	*p*	AUC	*p*	AUC	*p*	
1591		**0.833**	**0.014**	0.781	0.027	0.630	0.538	0.744	0.555	0.741	*P* < 0.0001	I
2118		**0.858**	**0.006**	**0.814**	**0.001**	0.681	1.00	0.708	1.00	0.761	0.002	I, II
2261		**0.958**	**0.029**	**0.961**	**0.005**	**0.886**	**0.006**	**0.970**	**0.02**	**0.945**	***P*** **< 0.0001**	I- IV, whole BC
2264		0.846	0.13	**0.885**	**0.023**	**0.856**	**0.017**	0.843	0.309	**0.857**	***P*** **< 0.0001**	II, III, whole BC
2337		**0.873**	***P*** **< 0.0001**	0.779	0.007	0.650	1.00	0.768	0.034	0.761	*P* < 0.0001	I
2379		**0.868**	***P*** **< 0.0001**	**0.844**	***P*** **< 0.0001**	0.725	0.60	0.772	0.016	0.796	*P* < 0.0001	I, II
2439		0.771	0.359	**0.881**	***P*** **< 0.0001**	0.757	0.114	**0.862**	***P*** **< 0.0001**	**0.821**	***p*** **< 0.0001**	II, IV, whole BC
2525		**0.84**	0.001	0.795	***P*** **< 0.0001**	0.773	0.035	**814**	**0.007**	**0.806**	***p*** **< 0.0001**	I, whole BC
2744		0.765	0.589	**0.82**	***P*** **< 0.0001**	0.643	1.00	0.738	0.136	0.739	*P* < 0.0001	II
3007		**0.875**	***P*** **< 0.0001**	**0.883**	***P*** **< 0.0001**	0.692	0.603	**0.84**	**0.004**	**0.818**	***P*** **< 0.0001**	I, II, IV, whole BC
3049		**0.827**	**0.009**	**0.815**	***P*** **< 0.0001**	0.587	1.00	0.692	0.286	0.721	*P* < 0.0001	I, II
3109		0.752	0.285	**0.839**	**0.001**	0.618	0.954	0.796	0.033	0.751	*P* < 0.0001	II
3195		**0.80**	**0.003**	0.723	0.05	0.755	0.347	0.778	0.102	0.763	*P* < 0.0001	I
3414		**0.816**	**0.016**	**0.837**	***P*** **< 0.0001**	0.576	1.00	0.778	0.043	0.747	*P* < 0.0001	I, II
3560		**1**	**0.003**	**1**	**0.018**	0.882	0.752	0.935	0.348	**0.944**	***P*** **< 0.0001**	whole BC
3719		**0.826**	**0.006**	**0.871**	**0.001**	0.621	1.00	0.745	0.035	0.762	*P* < 0.0001	I, II
3865		**0.985**	**0.001**	1	0.846	0.963	1.00	0.973	0.927	**0.977**	***P*** **< 0.0001**	I, whole BC

AUC value ≥0.8 and level of significant, *p* ≤ 0.05 at 95% CI were considered as double criteria to evaluate the distinguishing potential of a glycan between patients and normal NC. (AUC value vs diagnostic accuracy: 0.9–1 = “highly accurate”, 0.8–0.9 = “accurate”, 0.7–0.8 = “moderately accurate”, < 0.7 = “uninformative test”). The bold AUC and *p*-values indicate the glycans at the respective stage could fulfill both the criteria and thus were selected as candidate biomarkers

**Fig. 7:**
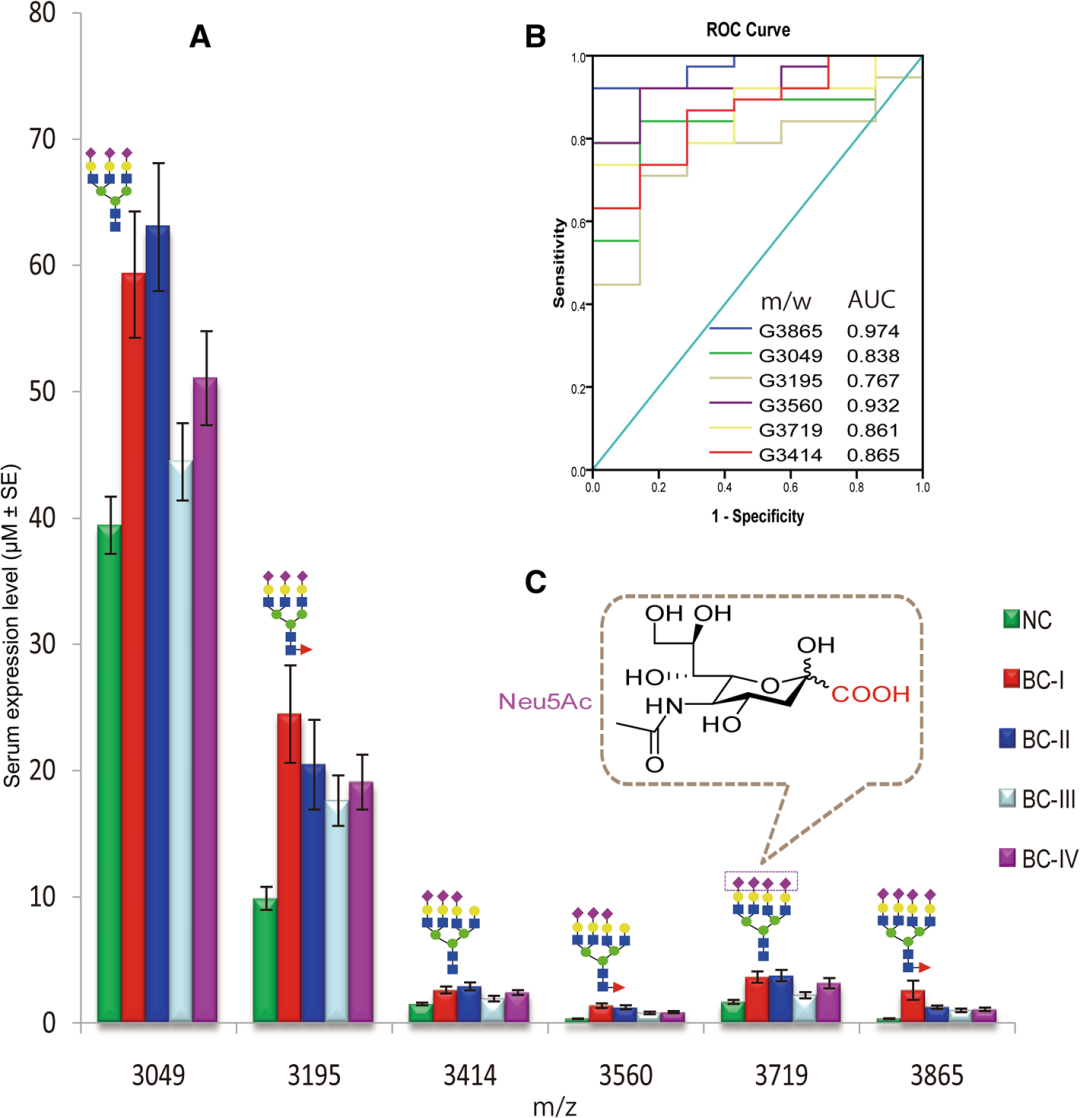
Serum expression abundance of hyperbranched and hypersialylated glyco-biomarkers. **a** Glycan expression level of the NC and BC stages of I-IV, **b** ROC curve and AUC value for their potential to distinguish the whole BC patients from NC, **c** chemical structure of sialic acid whose carboxyl group (COOH) has functional significance for cancer cell migration and metastasis

Dot plots and ROC curves for some of the identified candidate biomarkers of stage I, stage II, or all BC patients compared with NC are diagramed in Fig. 5. Strong correlation with the disease stages was observed in which a substantial increase in the glycans abundance was predominant at an early stage of the disease. Comparing with the control group, individual expression scores within the cancer groups have shown dispersed distribution. As clearly stated in Fig. 6, discriminating power of the candidate biomarkers that fulfilled both criteria (*p* ≤ 0.05 and AUC ≥0.8) was either specific to stage I and stage II or shared by multiple cancer stages. Among the 17 selected candidate biomarkers, 13 glycoforms for BC stage I patients, and 12 glycoforms for BC stage II patients could strongly predict and distinguish them from the control group. As an important observation, the most branched and sialylated structures have shown marked abundance in the serum of all cancer stages with a greater increase at an early stage (Fig. 7). Pair wise, the first (m/z 3049 vs 3195), the second (m/z 3414 vs 3560), and the third (m/z 3719 vs 3865) pairs are biosynthetically consistent, only differed by core-fucose moiety.

### Serum glycotyping pattern towards predicting early stage BC

**Fig. 8:**
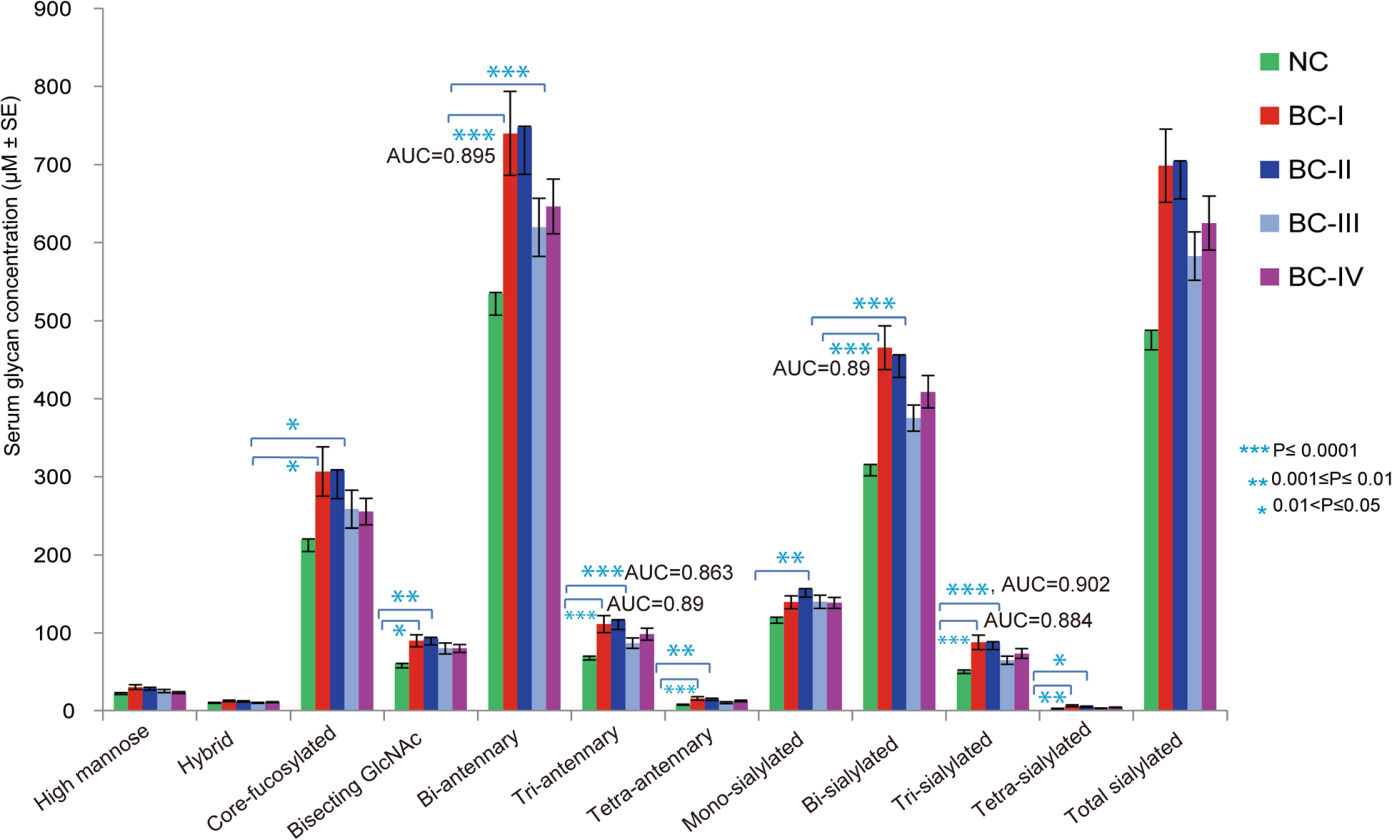
Serum abundance pattern of glycotypes sharing structural features comparing BC patients of stage I-IV with controls

Apart from individual serum *N*-glycan alteration, group of glyco-subclasses sharing certain structural features were also compared among different BC stages and controls. With no glyco-subclass altered significantly at stage III, we found statistically significant abundance in core-fucosylated, bisecting, bi-, tri-, and tetra-antennary, bi-, tri-, and tetra-sialylated *N*-glycans in the serum of stages I and II patients. From ROC analysis, greater diagnostic accuracy to predict early-stage BC was noticed in bi-antennary, bi-sialylated, tri-antennary, and tri-sialylated glycans (AUC = 0.895, .89, 0.863, 0.884, respectively) for stage I patients at *p* < 0.001. Moreover, tri-antennary, and tri-sialylated glycans demonstrated an AUC value of 0.88 and 0.902 to distinguish stage II patients from NC (Fig. 8). Larger abundance of these glycotypes associated with the early stages of the disease agrees with the aforementioned expression patterns of individual *N*-glycan biomarkers.

### IgG-derived *N*-glycans as BC biomarkers

**Fig. 9:**
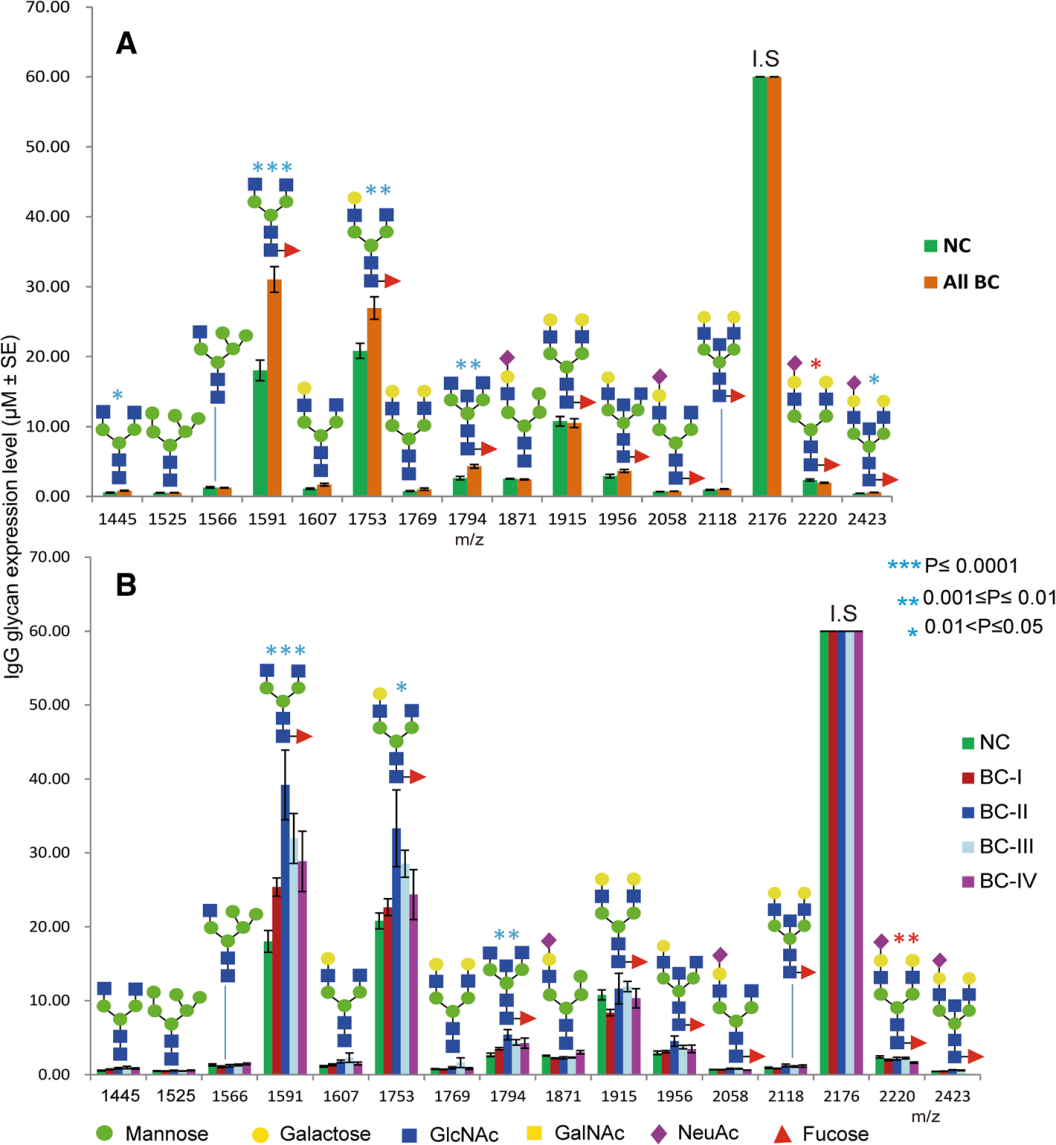
Expression levels and structures of *N*-glycans analyzed from purified IgG fraction. **a** All breast cancer patients vs normal controls, **b** Breast cancers stages vs normal controls. m/z 2176 is an internal standard (I.S), blue and red asterisk = significant increase and decrease, respectively in glycan level of BC group comparing with NC

To determine whether the altered glycosylation patterns of total serum *N*-glycome were associated with the immune system or not, we purposely focused on in-depth IgG *N*-glycome profiling of the study subjects. The result illustrated statistically significant elevation of 5 glycans (*m/z* 1445, 1591, 1753, 1794, and 2423) in the whole patient group (Fig. 9a), of which 3 glycans (*m/z* 1591, 1753, 1794) had substantial increase in the stage II patients comparing to the controls (Fig. 9b). Overall, 12 of the 15 detected IgG glycans belong to complex biantennary type within which core-fucosylation was noticed as a structural feature in 9 of them, including the 3 abundantly expressed in patients. Moreover, in the same way as in serum, only two IgG glycans (*m/z* 1915 and 2220) showed an overall slight reduction in the patient group.

**Fig. 10:**
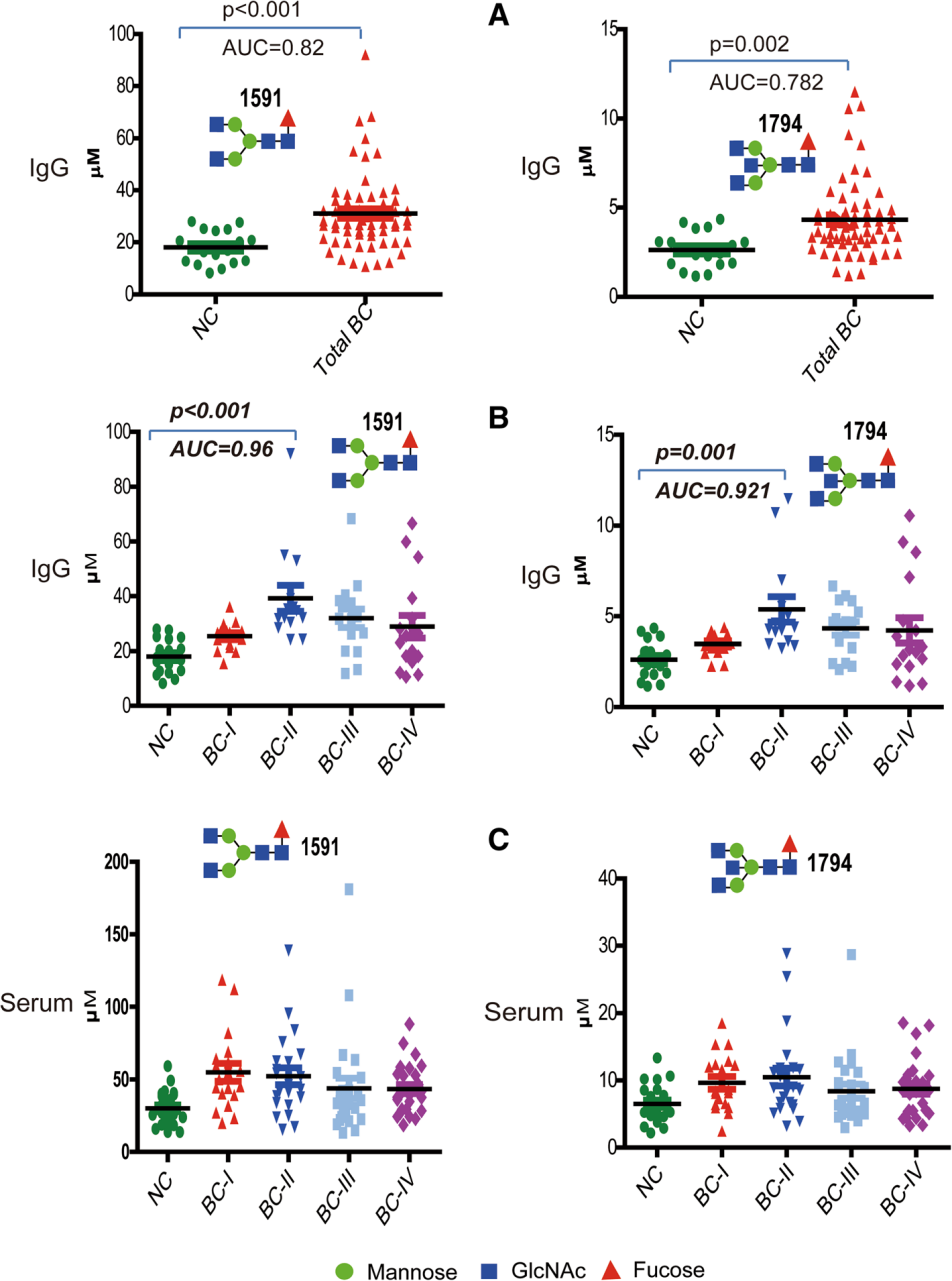
Dot plot illustrating two *N*-glycans highly expressed in IgG of BC patients. **a** Their distinguishing potential between whole BC patient group and NC, **b** Their high discrimination potential particularly between breast cancer stage II (BC-II) patients and NC. **c** Their similar serum expression pattern across the study groups

ROC test analysis indicated that, two IgG glycoforms (*m/z* 151 and 1794) could distinguish all BC patients and more strongly stage-II patients from the controls (AUC = 0.944 and 0.921, respectively; Fig. 10a, b). It should be noted that these glycans have shown quite similar patterns of abundance across the IgG and serum of all cancer stages and controls (Fig. 10b, c), reflecting that their carrier glycoprotein in the patients’ serum is mainly IgG. To determine IgG as a quantitatively reliable carrier of which circulatory *N*-glycans, ratio of *N*-glycan levels quantified from IgG fraction to that of whole serum were calculated and summarized in Additional file 4: Table S2. Thus, among the glycans whose larger proportion originated from IgG in BC patients include the glycans with m/z 1445 (53.25%), 1591 (64.76%), 1607 (41.72%), 1753 (59.41%), 1794 (46.64%), 1871 (53.24%), and 1915 (53.44%).

## Discussion

Apart from the cancer genome, deciphering alterations in protein glycosylation has been of critical importance not only to understand the molecular mechanisms of cancer progression, but also as a promising target for discovering novel diagnostic and therapeutic agents [6]. Despite significant advances in breast cancer health care system, the current diagnostic methods give procedural discomfort and suffer from lack of reliability and specificity to predict the disease at its early stage. Hence, novel serological biomarkers and drug targets are still needed to further improve breast cancer diagnosis and treatment [5]. The present study quantitatively profiled whole serum and IgG *N*-glycosylation alterations in Ethiopian breast cancer patients and matched controls. To the best of our knowledge, this is the first comprehensive report simultaneously addressing whole serum and purified IgG *N*-glycomics of breast cancer patients and more importantly employing study samples from native black African population whose glycosylation profile has not been studied elsewhere. We used a sensitive and an efficient glycoblotting method for glycan enrichment, after which we could identify and quantify a wide range of *N*-glycan structures by MALDI-TOF/MS analysis.

After careful data processing and analysis steps, 17 biosynthetically relevant complex type serum N-glycans (Table 3, Fig. 6) could specifically distinguish breast cancer patients from NC.

Structurally, majority of them belong to core-fucosylated, multiply branched and sialylated *N*-glycan types. With an overall quantitative up-regulation in all the breast cancer stages compared to NC, these serum glycans showed the highest abundance in the early stage (stage I and II) patients, making them promising diagnostic targets. Such a drastic and unidirectional quantitative shift over a wide-range of glycans was an unpredicted observation. ANOVA and ROC test analysis independently confirmed that these 17 *N*-glycans had high discriminating power to differentiate cancer stages from the healthy controls. Eight of these putative glyco-biomarkers could mutually distinguish (AUC = 0.8–1) both stage I and II patients from NC within which one glycan (*m/z* 2261) demonstrated high diagnostic performance in all stages of I-IV (Fig. 6).

Quantitative analysis of glyco-subclasses sharing the same structural residue (fucose, bisecting GlcNAc, antenna, and sialic acid) further intensified the predominant branching and sialylation features associated with early stage patients compared to controls. Among such glycotypes, it was observed that total bi-antennary and bi-sialylation for stage I, total tri-antennary and tri-sialylation for stage II strongly predicted and classified them from NC (Fig. 8). These results illustrate that aberrant serum glycan alterations in the breast cancer pathogenesis begin at an early stage of the disease and might be considered for non-invasive diagnostic biomarker identification. The current results are different from a previous study [19] that suggested 8 sialylated serum *N*-glycans (none of them had bisecting GlcNAc) as biomarkers associated with breast cancer. In the previous study, the predominant change (down-regulation of lower m/z and up-regulation of higher m/z glycans) of the suggested markers was observed in the late stage (stage IV) patients compared to controls. The candidate biomarkers identified in the present study, however, comprise asialo, bisecting, and hperbranched/hypersialylated glycans including only three of their reported glycan markers (m/z 2744, 3560, and 3719). Notably, our candidate biomarkers showed only an up-regulation pattern that was primarily noticed at the earlier stages (stage I or II) compared with the controls. As they used C18 column for glycan purification and permethylation (classical strategy)-based MALDI-TOF/MS analysis of relative intensities, it should be clear that the methodology and patient background of the two studies are quite different which makes their comparisons complicated. Thus, we hypothesize that the *N*-glycosylation changes associated with breast cancer progression in young black subjects seem to be unique and needs further attention.

It is well recognized that hypersialylation is a crucial feature in the progression of many cancers, whereby the negative charges in the terminal sialic acids of sugars interfere with epithelial cadherin-mediated cell–cell adhesion, enhancing the migratory and metastatic capacity of tumor cells [6, 38]. Interestingly, higher expressions of the glycosyltransferases responsible for core fucosylation (FUT8), branching (GnT-V), and sialylation (both α-2, 3 and α-2, 6 sialytransferases) have been associated with a greater potential for motility, invasion and metastasis in breast cancer [39–42]. One unexpected funding of our study was the up-regulation of a bisecting tetraantennary monosialylated glycan (m/z 3007) in the cancer groups as the *N*-acetylglucosaminyltransferase III (GnT-III) and its bisecting GlcNAc structures have been supposed to inhibit further branching in the biosynthesis pathway and suppress cancer metastasis [43]. Kinetic studies on GnT-V, another key enzyme for the synthesis of branched *N*-glycans, indicated that the enzyme can rarely use the bisecting GlcNAc as a substrate with a very low V_max_ value and produce branched bisecting structures [44]. Taking this into account, finding such a bisected and branched structure whose abundance was strongly associated with BC stages I, II, and IV may provide significant implications for a drastic up-regulation of the preceding steps in the biosynthetic machinery that could unusually satisfy the low affinity of the branching enzyme. Apart from the overall glycan alterations indicated earlier, such specific biochemical flux may in part reflect the aggressiveness and invasiveness of the disease. Additionally, this glycan seems to be unique to Ethiopian population as it was detected in neither of Japanese hepatocellular carcinoma patients up on simultaneous experimentation nor in any of our previous results of various diseases.

It is unclear and hardly reported what kind of carrier proteins are involved in breast cancer associated alterations of the serum *N*-glycan level. In a more detailed analysis, we purified the IgG fraction from the whole serum of all study participants and then integrated (Fig. 1) to our high-throughput glycoblotting method for subsequent glycan release and purification. MALDI-TOF/MS based quantitative analysis confirmed that many of the biantennary glycans including some of the suggested serum biomarkers (m/z 1591 and 2118) for early stage BC were originated from serum IgG (Additional file 4: Table S2). Two core-fucosylated and agalactosylated IgG glycans (m/z 1591 and 1794) were significantly up-regulated in the breast cancer patients, more specifically distinguishing patients with stage II breast cancer from NC. Their expression patterns in IgG and serum of controls and stages I-IV patients were found to be consistent (Fig. 10a**-**c), which proves that IgG is a reliable carrier for a larger proportion of their concentration in serum. Being one of the major *N*-glycosylated serum proteins and an essential part of the humoral immune system, IgG structure and function is importantly modulated by the abundance and types of glycoforms attached to its fragment crystallizable (Fc) region [45]. Increased core fucose in the IgG Fc domain has been shown to suppress its antibody-dependent cell-mediated cytotoxicity (ADCC) by decreasing its affinity towards cellular Fc receptors [46, 47]. On the other hand, increased IgG Fc galactose has recently been reported to enhance its complement-dependent cytotoxicity (CDC) activity via C1q binding [48, 49]. Based on our IgG glycomics result and these reports, the increased fucosylated and agalactosylated glycan structures observed in the cancer group appears to be a synergistic mechanism, allowing the tumor cells to escape from the immune system. In line with these results, increased IgG fucosylation (of non-sialylated glycans) and agalactosylation features have been previously reported in colorectal and gastric cancers [26, 27].

On the contrary, a recent study by Kawaguchi-Sakita N, et al., [25] has reported increased galactosylated IgG *N*-glycans associated with Japanese breast cancer patients. Accordingly, 7 galactosylated biantennary N-glycans when merged into a group showed significant abundance and predicted the probability of BC with a moderate accuracy whereas, unlike in the present study, no single IgG glycan could strongly distinguish BC patients from NC. Such dissimilarities can be caused not only by differences in analytical procedures but also differences in the patient background such as race, life style, age (as the majority of their study subjects were already in the menopausal state; over 50s y/o), all of which have been reported to affect the glycosylation pathway [50]. To ensure the reliability of our analytical procedure, quadruplicate of each sample was subjected to MALDI-TOF/MS analysis and the data for glycan levels were obtained from absolute quantification unlike many prior reports that had measured relative abundances. As such, the versatility of our glycoblotting method has been evidenced by its suitability for glycan purification from diverse samples (serum, cell lines, tissues, and CSF) [16–18, 29–34, 51] using only 10 μL of sample aliquots and overnight reaction. Apart from nonnegligible distinguishing features associated with BC, our IgG *N*-glycomics results clearly show that other unknown carrier proteins are greatly involved in the serum *N*-glycosylation alteration during breast cancer progression. Particularly, serum proteins that carry the hyperbranched and hypersialylated candidate glyco-biomarkers of BC are yet to be identified. Alpha-1-acid glycoprotein (AGP), an acute phase protein synthesized in the liver and secreted into the circulation, is believed to be one potential carrier for the bi-, tri-, or tetra- antennary complex type glycoforms. However, little is known about the association between breast cancer progression and AGP glycosylation pattern [4].

Many of the aberrant alterations observed in serum and IgG glycosylations of the early stage BC patients provide somehow different insight from previous reports which is most probably due to the relatively younger and black nature of the present study participants. In this regard, findings from various studies highlighted that young and black women are more likely to face an aggressive type of breast cancer, as it tends to have a poorer prognosis, a higher proliferative rate, a triple negative hormone receptor status, and a higher chance of **br**east **ca**ncer susceptibility genes (BRCA1 or BRCA2) mutations [52–55]. Based on the observations of the present study, being diagnostic biomarkers primarily at the early stage BC patients may raise a practical reliability doubt, especially in the context of African countries where awareness on cancer is low and patients are often diagnosed at advanced stages. Considering the advantage of early stage cancer markers to allow increased treatment options, this challenge can be alleviated by strengthening awareness creation and regular check-up strategies for breast cancer. In addition to the case-control groups, inter-and intra-subject variability of glycan levels were observed in the current study (much dispersed distribution has been shown within the cancer group). This suggests the potentials of protein glycosylation to be targeted for personalized medicine, as recently reported [56]. The current study, however, had limitation to reveal the carrier proteins of the identified biomarkers apart from IgG. Also, the suggested biomarkers from relatively small sample size demands further verification study on a larger sample size of well-matched case-control subjects before using them in the clinical area.

## Conclusions

In conclusion, this study comprehensively evaluated the total serum and IgG *N*-glycosylation signatures of native black cancer patients for the first time and identified novel *N*-glycan biomarkers showing strong diagnostic potential mainly at early stages of BC. Apart from the whole serum, our IgG focused *N*-glycome profiling provides evidence on BC-associated glycosylation alterations from the immune system perspective. Further study on direct identification of the intact glycoproteins using a glycoprteomics approach is expected to provide deeper understanding of specific biomarkers towards their clinical utility. Additionally, specific *N*-glycan profiling among the IgG subclasses of BC patients may lead to a focused diagnosis and therapy.

## Additional files

Additional file 1: **Figure S1.** Stacked-view MALDI-TOF Mass Spectra from large-scale serum *N*-glycomics of normal controls and BC patients. The raw mass spectra were further subjected to compositional analysis and structural elucidation. (TIF 8303 kb)

Additional file 2: **Figure S2.** Large-scale MALDI-TOF Mass Spectra of IgG *N*-glycans of normal controls and BC patients. The raw mass spectra were further subjected to compositional analysis and structural elucidation. (TIF 7665 kb)

Additional file 3: **Table S1.** List of serum *N*-glycans statistically differed between whole cancer patients and normal controls. All glycan peaks except m/z 1915 showed increased abundance (*p* ≤ 0.05) in the BC patients compared to NC. Peak numbers (peak #) are according to the list of detected glycans as shown in Table 2. (DOCX 15 kb)

Additional file 4: : **Table S2.** Percentage of IgG *N*-glycan level relative to their concentration in serum. NC = normal control group, BC = whole breast cancer group. Indicated in bold are those N-glycans whose quantity in IgG showed considerable contribution to their expression level in serum. (DOCX 11612 kb)

## Data Availability

The datasets used and analyzed during the current study are available from the corresponding author on reasonable request.

## Abbreviations

ADCC: Antibody-dependent cell-mediated cytotoxicity
AGP: Alpha-acid glycoprotein
ANOVA: Analysis of variance
AUC: Area under the curve
BC: Breast cancer
BMI: Body mass index
BRCA: Breast cancer susceptibility gene
CDC: Complement-dependent cytotoxicity
CSF: Cerebrospinal fluid
Fc: Fragment crystallizable
FUT8: Fucosyltransferase 8
GlcNAc: N-acetylglucosamine
GnT: N-acetylglucosaminyltransferase
IgG: Immunoglobulin G
MALDI-TOF/MS: Matrix-assisted laser desorption/ionization mass spectrometry
NC: Normal Controls
PNGase F: Peptide:N-Glycosidase F
ROC: Receiver operating characteristic
SDS-PAGE: Sodium dodecyl sulfate polyacrylamide gel electrophoresis
TNM: Tumor, Node, Metastasis
