# Supplementary figures and images for: Exploring serum and immunoglobulin G N-glycome as diagnostic biomarkers for early detection of breast cancer in Ethiopian women
Source: BMC Cancer. 2019 Jun 17;19:588. doi: 10.1186/s12885-019-5817-8 (PMC6580580; doi:10.1186/s12885-019-5817-8)

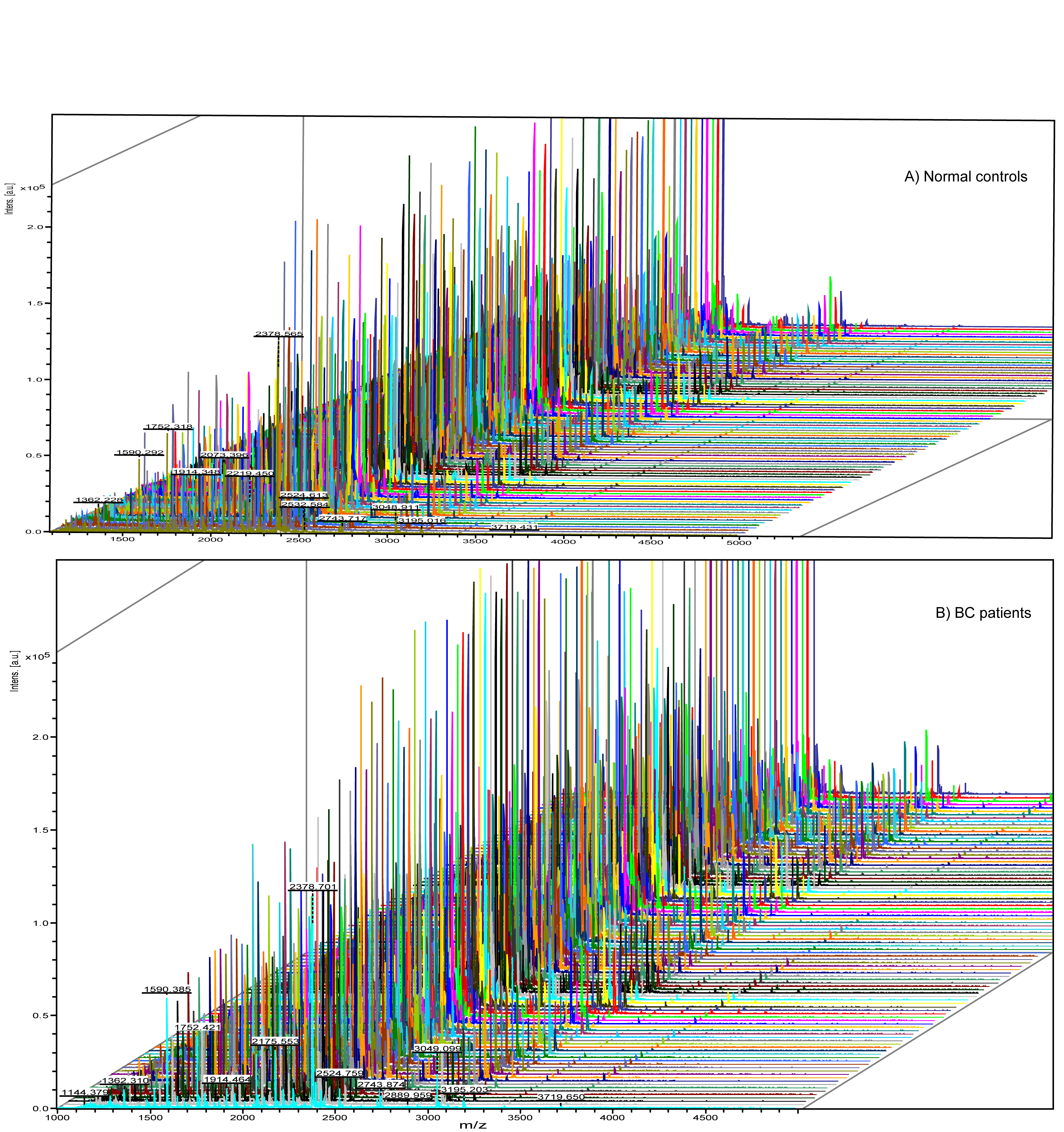

Supplement: Supplementary file 1 — Figure S1. Stacked-view MALDI-TOF Mass Spectra from large-scale serum N-glycomics of normal controls and BC patients. The raw mass spectra were further subjected to compositional analysis and structural elucidation. (TIF 8303 kb) [file 12885_2019_5817_MOESM1_ESM.tif]

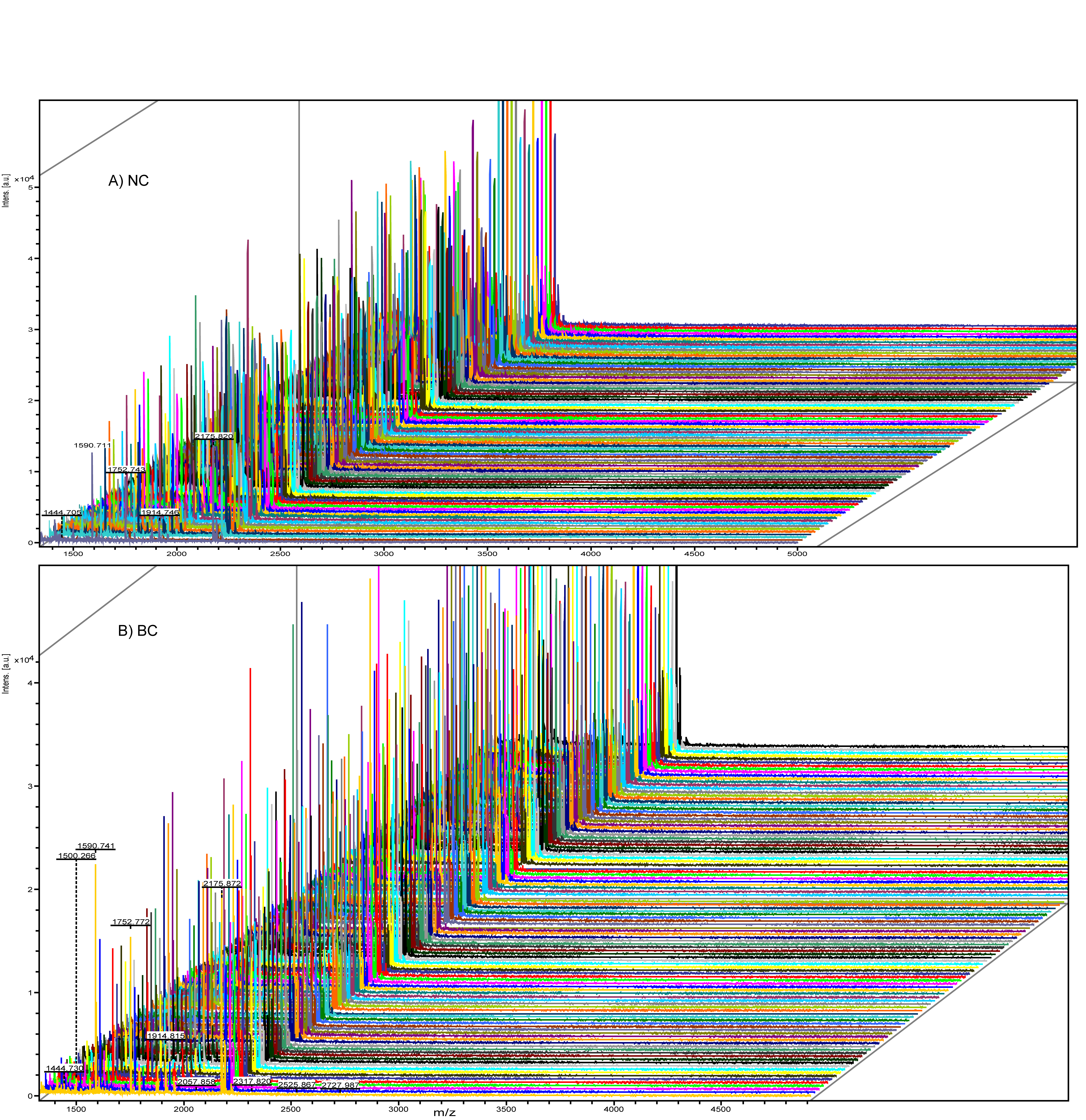

Supplement: Supplementary file 2 — Figure S2. Large-scale MALDI-TOF Mass Spectra of IgG N-glycans of normal controls and BC patients. The raw mass spectra were further subjected to compositional analysis and structural elucidation. (TIF 7665 kb) [file 12885_2019_5817_MOESM2_ESM.tif]
