# Supplementary material for: Exploring serum and immunoglobulin G N-glycome as diagnostic biomarkers for early detection of breast cancer in Ethiopian women
Source: BMC Cancer. 2019 Jun 17;19:588. doi: 10.1186/s12885-019-5817-8 (PMC6580580; doi:10.1186/s12885-019-5817-8)
Supplement: Supplementary file 3 — Table S1. List of serum N-glycans statistically differed between whole cancer patients and normal controls. All glycan peaks except m/z 1915 showed increased abundance (p ≤ 0.05) in the BC patients compared to NC. Peak numbers (peak #) are according to the list of detected glycans as shown in Table 2. (DOCX 15 kb) [file 12885_2019_5817_MOESM3_ESM.docx]

**Additional file 3: Table S1.** **List of serum *N*-glycans whose expression level statistically differed between whole cancer patients (BC) and healthy controls (NC).**

| Peak # | m/w |  | Serum expression level (AVG µM ± SE) | | |  | | |  | | |
| --- | --- | --- | --- | --- | --- | --- | --- | --- | --- | --- | --- |
|  |  |  | NC | BC |  | | | *p*-value (t-test) | | |  |
| 1 | 1362 | \| 6.65 ± 0.44 \| \| --- \| | | 8.20 ± 0.34 | | | 0.042 | | |  |  |
| 2 | 1445 | 0.79 ± 0.11 | | 1.55 ± 0.14 | | | *P*<0.0001 | | |  |  |
| 5 | 1591 | 30.07 ± 2.39 | | 47.94 ± 2.64 | | | *P*<0.0001 | | |  |  |
| 11 | 1794 | 6.53 ± 0.55 | | 9.26 ± 0.49 | | | 0.001 | | |  |  |
| 13 | 1855 | 0.55 ± 0.05 | | 0.70 ± 0.03 | | | *0.033* | | |  |  |
| 14 | 1871 | 4.20 ± 0.15 | | 4.57 ± 0.08 | | | *0.047* | | |  |  |
| 16 | 1915 | 25.54 ±1.62 | | 19.68 ± 1.34 | | | *0.048* | | |  |  |
| 18 | 2011 | 3.57 ± 0.17 | | 4.53 ± 0.17 | | | *0.009* | | |  |  |
| 19 | 2033 | 2.20 ± 0.11 | | 2.63 ± 0.06 | | | *0.002* | | |  |  |
| 21 | 2074 | 48.64 ± 2.22 | | 57.49 ± 1.58 | | | *0.011* | | |  |  |
| 22 | 2118 | 7.21 ± 0.66 | | 10.92 ± 0.52 | | | *0.002* | | |  |  |
| 25 | 2261 | 1.43 ± 0.11 | | 3.50 ± 0.18 | | | *P*<0.0001 | | |  |  |
| 26 | 2264 | 1.14 ± 0.09 | | 2.10 ± 0.10 | | | *P*<0.0001 | | |  |  |
| 27 | 2277 | 4.60 ± 0.33 | | 6.29 ± 0.24 | | | *0.001* | | |  |  |
| 28 | 2337 | 4.65 ± 0.20 | | 6.10 ± 0.19 | | | *P*<0.0001 | | |  |  |
| 29 | 2379 | 269.88 ± 11.06 | | 358.85 ± 9.81 | | | *P*<0.0001 | | |  |  |
| 30 | 2423 | 14.09 ± 1.34 | | 20.63 ± 0.73 | | | *P*<0.0001 | | |  |  |
| 31 | 2439 | 4.12 ± 0.20 | | 6.01 ± 0.20 | | | *P*<0.0001 | | |  |  |
| 32 | 2484 | 2.53 ± 0.12 | | 2.99 ± 0.10 | | | *0.008* | | |  |  |
| 33 | 2521 | 1.86 ± 0.09 | | 2.32 ± 0.07 | | | *P*<0.0001 | | |  |  |
| 34 | 2525 | 22.11 ± 1.07 | | 31.46 ± 0.98 | | | *P*<0.0001 | | |  |  |
| 35 | 2582 | 0.71 ± 0.05 | | 1.13 ± 0.05 | | | *0.001* | | |  |  |
| 36 | 2584 | 1.00 ± 0.07 | | 1.57 ±0.05 | | | *P*<0.0001 | | |  |  |
| 37 | 2728 | 7.89 ± 0.62 | | 11.81 ± 0.51 | | | *P*<0.0001 | | |  |  |
| 38 | 2744 | 8.49 ± 0.43 | | 11.58 ± 0.44 | | | *P*<0.0001 | | |  |  |
| 39 | 2890 | 1.60 ± 0.10 | | 2.80 ± 0.16 | | | *P*<0.0001 | | |  |  |
| 40 | 3007 | 0.46 ± 0.04 | | 0.77 ± 0.03 | | | *P*<0.0001 | | |  |  |
| 41 | 3049 | 39.42 ± 2.27 | | 53.86 ± 2.10 | | | *P*<0.0001 | | |  |  |
| 42 | 3109 | 1.25 ± 0.07 | | 1.98 ± 0.10 | | | *P*<0.0001 | | |  |  |
| 43 | 3195 | 9.88 ± 0.91 | | 20.10 ± 1.36 | | | *P*<0.0001 | | |  |  |
| 44 | 3414 | 1.50 ± 0.11 | | 2.44 ± 0.12 | | | *P*<0.0001 | | |  |  |
| 45 | 3560 | 0.33 ± 0.03 | | 1.00 ± 0.07 | | | *P*<0.0001 | | |  |  |
| 46 | 3719 | 1.66 ± 0.16 | | 3.16 ± 0.20 | | | *P*<0.0001 | | |  |  |
| 47 | 3865 | 0.33 ± 0.03 | | 1.31 ± 0.14 | | | *P*<0.0001 | | |  |  |

All glycan peaks except m/z 1915 showed increased abundance (p≤0.05) in the BC patients compared to NC.
