# Supplementary material for: Exploring serum and immunoglobulin G N-glycome as diagnostic biomarkers for early detection of breast cancer in Ethiopian women
Source: BMC Cancer. 2019 Jun 17;19:588. doi: 10.1186/s12885-019-5817-8 (PMC6580580; doi:10.1186/s12885-019-5817-8)
Supplement: Supplementary file 4 — : Table S2. Percentage of IgG N-glycan level relative to their concentration in serum. NC = normal control group, BC = whole breast cancer group. Indicated in bold are those N-glycans whose quantity in IgG showed considerable contribution to their expression level in serum. (DOCX 11612 kb) [file 12885_2019_5817_MOESM4_ESM.docx]

**Additional file 4: Table S2.** **Percentage of IgG *N*-glycan level relative to their concentration in serum.**

|  |  | | Glycan concentration (µM) | | | |  | % Contributed by IgG | |
| --- | --- | --- | --- | --- | --- | --- | --- | --- | --- |
| m/z | Glycan |  | Serum  (NC) | Serum (BC) | IgG (NC) | IgG (BC) |  | % (NC) | % (BC) |
| 1445 | 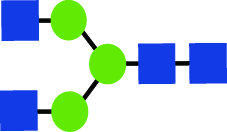 | 0.79 | | 1.55 | 0.56 | 0.83 | | **70.87** | **53.25** |
| 1525 | 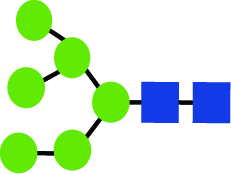 | 6.88 | | 8.17 | 0.52 | 0.54 | | 7.57 | 6.56 |
| 1566 | 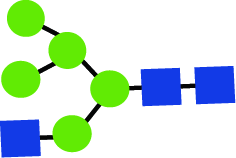 | 0.88 | | 1.03 | 1.29 | 1.25 | | **146.04** | **121.10** |
| 1591 | 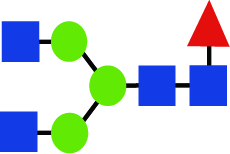 | 30.07 | | 47.94 | 18.03 | 31.05 | | **59.94** | **64.76** |
| 1607 | 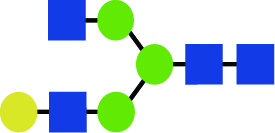 | 3.41 | | 4.05 | 1.10 | 1.69 | | **32.12** | **41.72** |
| 1753 | 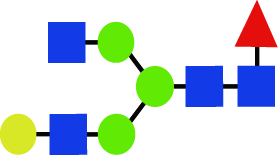 | 42.26 | | 45.38 | 20.82 | 26.96 | | **49.26** | **59.41** |
| 1769 | 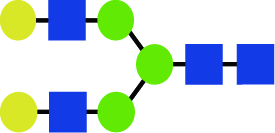 | 5.19 | | 5.76 | 0.77 | 1.02 | | 14.89 | 17.76 |
| 1794 | 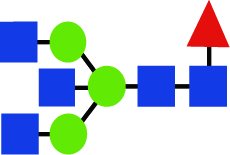 | 6.53 | | 9.26 | 2.62 | 4.32 | | **40.19** | **46.64** |
| 1871 | 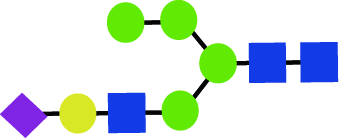 | 4.20 | | 4.57 | 2.53 | 2.43 | | **60.21** | **53.24** |
| 1915 | 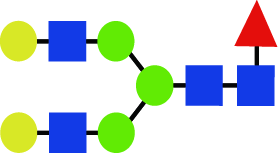 | 25.54 | | 19.68 | 10.79 | 10.52 | | **42.23** | **53.44** |
| 1956 | 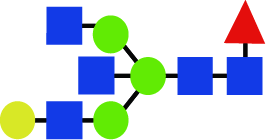 | 9.13 | | 10.36 | 2.92 | 3.66 | | **31.94** | **35.29** |
| 2058 | 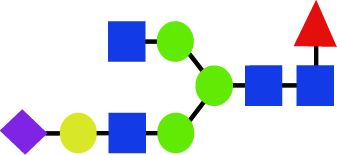 | 3.02 | | 3.29 | 0.69 | 0.75 | | 22.71 | 22.85 |
| 2118 | 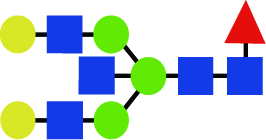 | 7.21 | | 10.92 | 0.94 | 1.06 | | 13.07 | 9.69 |
| 2220 | 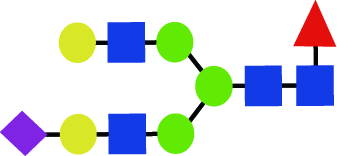 | 28.54 | | 28.52 | 2.34 | 1.96 | | 8.21 | 6.86 |
| 2423 | 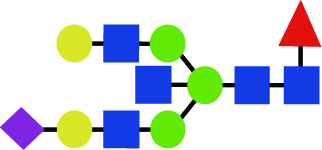 | 14.09 | | 20.63 | 0.44 | 0.57 | | 3.13 | 2.77 |

NC = normal control group, BC = whole breast cancer group. Indicated in bold are those *N*-gylcans whose quantity in IgG showed considerable contribution to their expression level in serum.
